# Gastrointestinal stromal tumors and shock

**DOI:** 10.4103/0974-2700.55344

**Published:** 2009

**Authors:** Karim Ibn Majdoub Hassani, Fatim Zahra Zahid, Abdelmalek Ousadden, Khalid Mazaz, Khalid Ait Taleb

**Affiliations:** Department of General Surgery, Universitet Hospital, Hassan II, Fes, Morocco

**Keywords:** Emergency surgery, gastrointestinal stromal tumor, hemorrhagic shock, upper gastrointestinal bleeding

## Abstract

Gastrointestinal stromal tumors (GIST) are the most common mesenchymal tumor of the gastrointestinal tract. Clinically, they are associated with nonspecific symptoms, but some patients can present gastrointestinal bleeding with shock. We report two cases of GIST of the small bowel, revelated by hemorrhagic shock secondary to acute bleeding, succesfully treated by emergency surgery.

## INTRODUCTION

Gastrointestinal stromal tumors (GIST) are the most common mesenchymal tumors of the gastrointestinal tract. They can occur anywhere along the gastrointestinal tract but are most common in the stomach and small bowel. They are characterized by a remarkable cellular variability and their malignant potential is sometimes difficult to predict. Clinically, GIST are associated with nonspecific symptoms, but some patients can present gastrointestinal bleeding with shock. Emergency surgery after resuscitation is the treatment of choice for severely hemorrhagic GIST. Prognosis of GIST after surgical treatment is influenced by completeness of primary resection and tumor malignant potential. Adjuvant treatment (Imatinib) should be advocated for patients with either a high-grade GIST or after incomplete primary surgical treatment.

## CASE REPORTS

### Case 1

**Figure 1:**
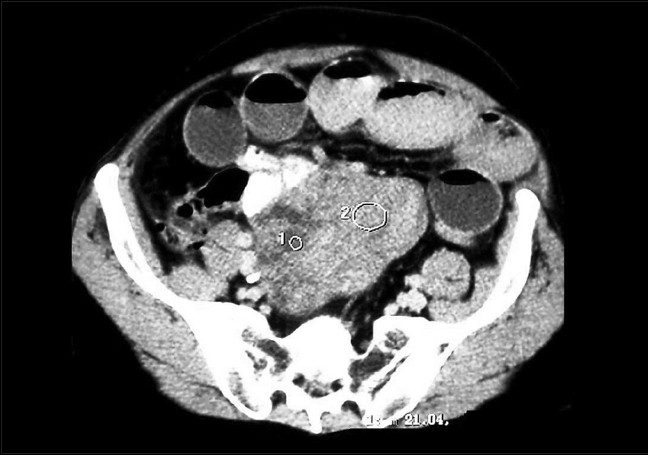
Computerized tomography scan revealing a solid hyperdense-enhancing mass (diameter of 12 × 11 × 8 cm) of the small bowel, with some necrotic components

**Figure 2:**
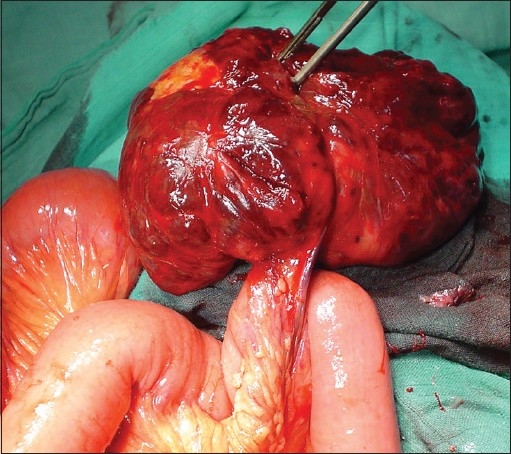
Peroperatory finding tumor of the small bowel at 90 cm of the ileo-ceacal junction

**Figure 3:**
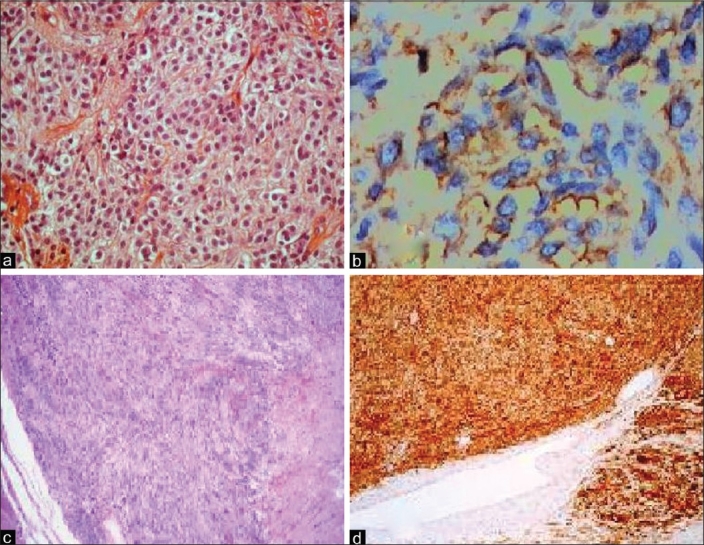
Histological examination and immunohistochemical (IHC) profiles of the resected tumors; (a) Epithelioid cell proliferation suggests gastrointestinal stromal tumors (H and E, ×400); (b) IHC staining positive for CD-117 (brown color); (c) Predominantly spindle cells (H and E, ×100); (d) IHC showing a diffuse positive signal for CD-34 (H and E, ×100)

A 54-year-old man was admitted to the emergency department because of acute abdominal pain. He had a 3-month history of abdominal pain, bloating, anemia and three episodes of melena that resolved spontaneously. He was not using any specific medication and his medical history did not suggest any major disease. The physical examination showed a conscious patient hemodynamically stable whose temperature was 37°C, whose blood pressure was 130/80 mmHg and who had a good nutritional status and slightly discolored conjunctives. Abdominal examination showed a tenderness and a mobile hypogastric mass. The hemoglobin level was 8.2 g/dl and reduced hematocrit (24.9%) and iron level suggested chronic bleeding. The platelet count was 260,000/μL, white blood cell count was 10200/mm^3^ (79% neutrophils), blood urea was 0.25 g/L and the creatinine level was 10 mg/L. Liver enzymes and hemostasis laboratory data were normal. The patient was hospitalized in the gastroenterology department for etiological investigation. The upper digestive endoscopy and colonoscopy were normal. No gastric or colic lesions were found as an explanation of the melena. An ultrasound and abdominal computerized tomography (CT) were realized to explore the abdominal mass, which showed a large heterogeneous tumor of the small bowel (diameter of 12 × 11 × 8 cm) [Figure 1]; thus, the decision to refer the patient for surgery. But, during his hospitalization, before his transfer to the surgery department, the patient developed a fulminant gastrointestinal hemorrhage represented by a severe melena, with a hemorrhagic shock, sudden hypotension and shortness of breath, a profuse sweating and a tachycardia with a heart rate of 117 beats/min. He was admitted to the intensive care unit (ICU) with a swift assessment of his airway, breathing and circulation. Resuscitation was begun, after central intravenous access, with saline and conventional crystalloid solution and then transfusion of the red blood cells (6 units) was performed after blood grouping and cross-matching. An emergency exploratory laparotomy was performed under general anesthesia after hemodynamic stability and satisfactory control coagulogram (hemoglobin level 11.6 g/dl). Surgical exploration showed a pediculated tumor of the small bowel at 90 cm of the ileo-ceacal junction [Figure 2], there was no hemoperitoneum and no other tumoral localizations were noted. A complete tumor resection with intact pseudocapsule and more than 1 cm of tumor-clear margins was performed. The patient recovered well without postoperative complications. Pathological examination revealed a malignant GIST. The tumor measured more than 12 cm in dimension and the mitotic rate was 20/50 high-power fields, with abundant tumor necroses. The tumor was focally positive for KIT (CD117) [Figure 3a and b].

### Case 2

**Figure 4:**
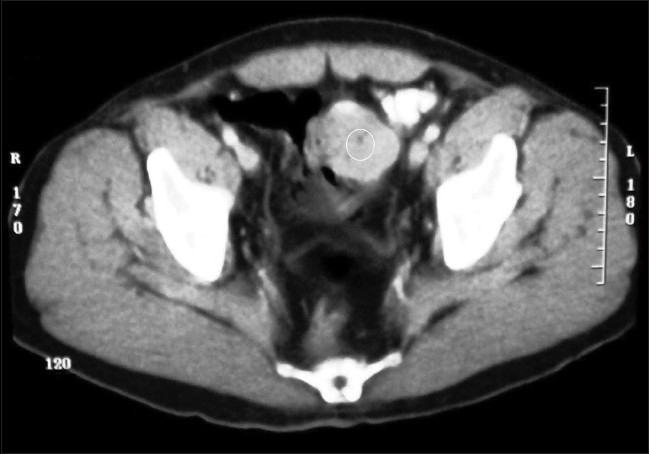
Computerized tomography showing a hyperdense-enhancing tumor

A 63-year-old man was admitted in the emergency room of the University Hospital of Hassan II with acute abdominal pain, hypotension, melena and severe anemia. His medical history revealed recurrent melena and upper abdominal discomfort over the last 4 months and did not suggest any major disease except hypertension treated for 3 years. He had no prior history of abdominal surgery or trauma. The physical examination revealed a conscious man with severe cutaneous paleness, a blood pressure of 90/60 mmHg, profuse sweating and a tachycardia with a weak and rapid pulse rate of 120 beats/min. There was no fever and there was minimal tenderness in the hypogastria at the abdominal examination. Laboratory data on admission showed a white blood cell count of 7,900/mm^3^, a hematocrit of 19% and a serum hemoglobin concentration of 6.4 g/dl, with a normal blood platelet level (390,000/mm^3^). Hemostasis laboratory data was normal. The patient was admitted to the ICU and was provided, by taking a central venous line (peripherally inserted central catheter) introduction of a nasogastric tube and urinary catheter, initial resuscitation by physiological serum and macromolecules and then transfusion with 8 units of red blood cells. After hemodynamic stability, a gastroscopy was performed, which did not demonstrate any gastric lesion responsible for this hemorrhage. No lesions or points of bleeding were found at the colonoscopy. But, the abdominal ultrasonography (US) realized after endoscopic explorations revealed the presence of a tissular mass in the left iliac fossa and the CT confirmed results of US and showed a solid tumor measuring (6 × 4.5 cm), probably a GIST of the small bowel [Figure 4].

**Figure 5:**
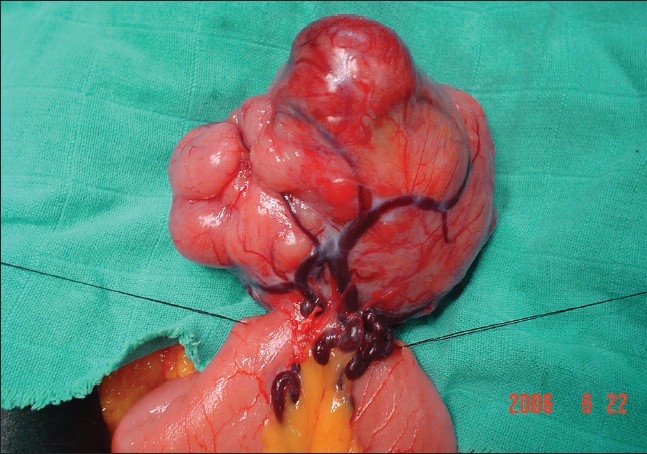
Surgical exploration notes a tumor of the terminal ileum

Even if hemodynamic parameters were normalized after resuscitation and transfusion, and the hemoglobin control level was 10.9 g/dl, and because of the persistence of the severe melena, it was indispensable to shift the patient to the operating room for an emergency surgery to control the source of bleeding. A laparotomy was performed. Exploration noted a tumor of the terminal ileum [Figure 5] and no other tumoral localization was found. A small bowel resection removing the tumor was performed with terminoterminal anastomosis. Postoperatively, there were no complications and the patient made an excellent recovery. Histological examination described a stromal tumor with free margins. Immunohistochemical markers confirmed the diagnosis of GIST (CD 34 positive, CD 117 negative, AML negative) [Figure 3 c and d]. The re-evaluation, performed 6 months and 2 years later, showed complete recovery, without any sign of abdominal CT recurrence of the disease.

## DISCUSSION

GIST are the most common mesenchymal tumors of the gastrointestinal tract.[1] The common sites of location are, in order, the stomach (60-70%), the small intestine (20-30%), the rectum and the colon (5%), the esophagus and a small percent may be located elsewhere in the abdominal cavity (<5%).[2] Clinically, GIST are associated with nonspecific symptoms. No physical findings specifically suggest the presence of a GIST. The main manifestation of GIST is acute or chronic upper gastrointestinal hemorrhage (61%).[3] Many GIST are discovered incidentally during operation, abdominal imaging or endoscopy. Tumors found incidentally are usually small, with a mean diameter of 1.5 cm, and carry a better prognosis.[4] Although extraluminal in origin, GIST may ulcerate through the overlying mucosa.[5] GIST of the small bowel typically present with vague symptoms, including abdominal pain, anemia because of a chronic bleeding, weight loss or gastrointestinal hemorrhage. A severe hemorrhage can be complicated by a hemorrhagic shock, with necessity of fluid replacement and transfusions, and sometimes necessity of an emergency surgery to control the source of the bleeding, which is the case of our two patients. Hemorrhagic shock is defined as a condition of reduced perfusion of vital organs leading to inadequate delivery of oxygen and nutrients necessary for normal tissue and cellular function. In patients with suspected GIST with severe bleeding and hemorrhagic shock, resuscitation, fluid management and blood transfusion are indispensable for normalization of hemodynamic parameters and restoring hemodynamic stability, first to confirm the diagnosis of the tumor and its localization by endoscopic and radiological modalities, and in the second, to prepare the patient for adequate surgery in good conditions. In unstable or unresponsive hemorrhagic shock, surgical treatment is mandatory as soon as possible to control the source of bleeding. The upper digestive endoscopy and colonoscopy must be performed in patients with acute gastrointestinal bleeding, to find gastric or colic lesions explaining the hemorrhage. US is the initial imaging modality when evaluating abdominal mass or nonspecific abdominal symptoms; contrast-enhanced is the imaging modality of choice to characterize an abdominal mass as well as to evaluate its extent and the presence or absence of metastasis. Typically, GIST are solid, hyper dense-enhancing masses on CT, and are typically exophytic, but the origin may be difficult to identify when the mass is very large. However, large GIST (>10 cm) are often more complex because of necrotic, hemorrhagic or degenerating components. When a submucosal tumor is found incidentally during upper endoscopy or colonoscopy, the extraluminal extent of disease should be evaluated using CT.[6] The diagnosis of GIST is secured by immunohistochemical staining for the tyrosine kinase receptor KIT (CD 117), which highlights the presence of interstitial cells of Cajal (ICC). CD 117 expression also differentiates GIST from true leiomyomas and gastric schwannomas, which are consistently negative for CD 117. About 95% of GIST are positive for KIT (CD117), 60-70% for CD34, 30-40% for smooth muscle actin, 5% for S-100 protein, 1-2% for desmin and 1-2% for keratin.[7] In general, tumor size and mitotic index are accepted as two independent prognostic factors for diagnosis.[8] For GIST, prognostic markers that include size larger than 5 cm, mitotic rate >5/50, high-power fields, tumor necrosis and a Ki-67 (MIB-1) index ≥10% all are associated with malignancy and high mortality.[9,10] Surgery is the mainstay of therapy for GIST when the primary lesion is deemed resectable. The goal is complete gross resection with an intact pseudocapsule and negative microscopic margins. The tumor must be handled with care to prevent intraabdominal rupture and dissemination. Tumor rupture before or during resection is a predictor of poor outcome.[11] Lymphatic spread of GIST is uncommon and therefore a formal lymph node dissection is not standard surgical management. Consequently, complete surgical resection of the primary tumor is the most definitive treatment.[12] We believe that emergency surgery is the treatment of choice for severely hemorrhagic GIST with shock, especially in the small bowel localizations. In the present cases, complete resection was achieved with more than 2 cm of tumor-clear margins, which is the recommended approach for GIST resection.[13–15] For both large tumors and poorly positioned small GIST that are considered marginally resectable on technical grounds, neoadjuvant imatinib is recommended. Patients with primary localized GIST whose tumors are deemed unresectable should also start imatinib.

## CONCLUSION

GIST are a subset of gastrointestinal mesenchymal tumors, although relatively rare in absolute terms. The main manifestation of GIST is acute or chronic gastrointestinal hemorrhage, especially in the small bowel localizations, and sometimes this bleeding can be associated with hemorrhagic shock. Emergency surgery after resuscitation, fluid management and blood transfusion is the treatment of choice in these cases. Complete surgical resection of the primary tumor is the most definitive treatment. Prognosis of GIST is influenced by completeness of primary resection and tumor malignant potential. Adjuvant treatment (imatinib) should be advocated for patients with either a high-grade GIST or after incomplete primary surgical treatment.
